# On Chronic Inflammation of the Testis; with a Series of Cases Illustrating the Subject

**Published:** 1831-04-01

**Authors:** 


### XVII.

### ST. GEORGE'S HOSPITAL.

### CHRONIC INFLAMMATION OF THE TESTIS.

The diseases of the testis have lately attracted much attention, and the able work of Sir Astley Cooper will communicate information on the subject to a wide circle of professional readers. There are very few points of surgery or of physic, however assiduously they may have been studied by even the ablest men, which are altogether incapable of further elucidation. The high road to truth is the knowledge of facts, and well it is for her when those facts are carefully studied and accurately recorded. In the investigation of disease, we peruse individual cases in the great book of nature. Cases then are the language of disease, symptoms are its alphabet, and the best and wisest practitioner is he who can decipher the characters and read the page, without the deceitful help of the glass of theory, or the gloss of error. In former times we had chiefly theoretical works upon medicine, and for this plain reason, that dissection did not correct the misconceptions of mere observation and experience. We are revolutionized now, if you will we are reformed. The diffusion of the knowledge of general and special healthy anatomy, and the yet more modern cultivation of morbid anatomy have constituted a new epoch, and formed, as it were, new views and new men. Out of evil cometh good, out of good, evil. We are not so much advanced in the treatment, as we are in the knowledge of the nature of diseases; some doubt if our therapeutics are as good as our forefathers'. But this is not the business of the present report, and we return to the point from which we started; that the study of individual cases will form, and alone will form, the good practitioner, whether in surgery or physic. It is the object of reporting to convey, in some sort and fashion, the experience to be gained in a hospital to those who have not access to such institutions. With the utmost circumspection in observing and fidelity in recording, this object can only be imperfectly attained; without these qualifications it cannot be attained at all. We have endeavoured, and we shall endeavour, to render the cases which we are the means of conveying to the public as authentic as our poor abilities will permit, and to any thing beyond this most necessary qualification we make no pretence.

Chronic inflammation of the testis may arise in two ways; it may be the sequela of acute inflammation, or it may steal on without any severe symptoms in the first instance. In the former variety, the nature of the case is usually made out with little or no difficulty, indeed the experienced surgeon will detect it in an instant. It is not always so with the latter; obscurity frequently hangs on the diagnosis, and the doubts of the surgeon are occasionally satisfied only by a sort of ex-post-facto inference, by the effects of mercury. Sir Astley Cooper appears to consider the two forms of chronic inflammation of the testis as essentially the same. We cannot but suspect that there is a greater difference between them than the able Baronet allows. In the chronic inflammation of the testis following the acute, the epididymis and vas deferens are more peculiarly affected, there is a less disposition to the tubercular formations in the testis, and the affection is more completely under the control of mercury, than we see in the "chronic inflammation," or yellow tubercular disease, not preceded by active inflammation. Such, at least, are the impressions produced on our mind by the facts which we have witnessed, but the observations of a young reporter are as dust in the balance, when weighed with the opinions of experienced surgeons.

We might detail many cases of hernia humoralis, but most practitioners must have witnessed examples of the affection, and we need not detain our readers by relating any. The following facts will serve our purpose, as they shew the condition of the vas deferens and epididymis, left after acute attacks of inflammation.

Case 1.—*Acute Inflammation of each Vas Deferens and Epididymis successively.*

A man was admitted under Mr. Keate with retention of urine, from stricture of long standing, which for three weeks previously had been impermeable to any instrument. Small bougies were passed through the stricture, but a false passage appeared to exist. The hip-bath, anodynes, and purges were employed, and the size of the bougie was gradually increased. On the 18th of Feb. of the present year a bougie was introduced. On the 20th, he was seized with thickening, induration, and extreme tenderness of the vas deferens, as far as it could be felt, and of the epididymis on the left side. He had no affection of the testis previously to this.

*Hirud.* vj. *Cat. lini. H. sennæ. Coxarium.*

On the 21st, he felt slight pain in the other testis, and, on the 22d, the bougie was passed again. In the afternoon there was enlargement, induration, and excessive tenderness in the vas deferens and epididymis on the right side. Next day these symptoms were increased; there was fever, and the urine was loaded with salts.

*C.c. lumbis, ad* ℥vj. *H. sal. c. Pulv. Ip. c.* gr. v. 6*tis horis.*

He was relieved by the cupping. On the 26th, the swelling and pain on the left side were diminished, and a little so on the right. After this, the pain and enlargement gradually diminished on both sides; but, on the 8th of March, there was still induration both of the epididymis and vas deferens, and some tenderness on pressure. The body of the testis was apparently unaffected on both sides. It is curious that the patient now became subject to intermittent fever, connected evidently with the state of the urethra.

Case 2.—*Suppression of Gonorrhœa—Inflammation of the Vas Deferens, Epididymis, and Body of the Testis.*

An out-patient had gonorrhœa, which had existed for some weeks; the scalding was much abated, the discharge thick, but less. On a Saturday night, after exposure to a cold wind in his occupation of street-sweeping, the discharge and scalding suddenly ceased, and he found the right testicle grow painful first, and then become enlarged. On Monday he applied at the hospital. The vas deferens was nearly as thick as one's little finger, hard, exquisitely painful when touched; the epididymis was in the same state; the body of the testis a little enlarged and tender; pain in the loins; little fever; no urethral discharge or scalding.

*To bathe the penis often in warm water—haust. sennæ stat.—C.c. lumbis ad* ℥vj.

Two more cuppings were required, and salines, with antimony, &c. The pain and swelling of the body of the testis yielded first, then the vas deferens resumed its natural state; but, on the 8th March, there were still tenderness and induration of the epididymis, although they were less than before. Two or three days previously to this, the discharge had re-appeared in the urethra, with slight scalding. The treatment now consisted in the application of camphorated mercurial ointment to the scrotum, support of the parts, and the administration of infusion of salts and roses.

Here were two cases of acute inflammation, chiefly of the vas deferens and epididymis, consecutive to irritation or inflammation in the urethra. After the violence of the inflammation was subdued, there remained in each induration and enlargement of the epididymis and vas deferens, requiring a longer or shorter time, and the employment of certain means for their complete removal. The affection of the body of the testis in each was slight, and in each soon passed away. We will take the same affection under other circumstances.

CASE 3. *Rupia — Griping, &c. from Mercury—Inflammation of one Testicle.*

A man had been for some time in the hospital, under Mr. Hawkins, for rupia, the exciting cause of which was cold. He had also ulcerated sore throat, and rupia-ulcerations of the conjunctiva lining the palpebræ of both eyes. He was put upon sarsaparilla, but obliged to omit it on account of fever. He resumed it, along with blue pill, but this had only been taken five days when it griped him and made his mouth sore. He also complained of pain in the right testis, and on examination the scrotal portion of the vas deferens was found indurated, swollen, and painful; the epididymis was the same. He said that previous to this attack he had been subject to slight induration of the parts affected. Under leeches, the omission of the mercury, the application of a stimulating lotion, and persistance with the sarsaparilla, the affection readily subsided and left the testis as before.

It is most probable that, in his instance, the inflammation of the epididymis and vas deferens was occasioned by the mercury, as it accompanied, with singular correspondence, other well known effects of that drug. If the supposition be correct, the fact is another instance of a disease producible and curable by mercury. In the next case we see a more ulterior stage of the inflammation of the vas deferens and epididymis.

CASE 4. *Chronic following Acute Enlargement of the Vas Deferens and Epididymis.*

James Harris, æt. 21, was admitted Sept. 8, 1830, under Mr. Babington. In the epididymis of the left testis was a solid enlargement, about the size of an almond, or bigger, chiefly seated in the cauda and body of the epididymis. The vas deferens was indurated and thickened as high as it could be traced, and both it and the epididymis were tender upon pressure. Thick yellow discharge from the urethra without scalding—health very good.

Six or seven weeks previously he had caught a gonorrhœa. About four weeks previously, being exposed to cold, the urethral discharge ceased, and inflammation of the testis suddenly set in. He did nothing for it, and in the course of three weeks the swelling had in great measure subsided, leaving the thickening of the epididymis. At this time the urethral discharge returned.

*Recumbent posture — Ung. hyd. fort. c. Camph. testi. Calomel*, gr. iij., *o. n. H. sennæ, o. m.*

On the 14th, the mouth was slightly affected, the enlargement of the epididymis was diminishing, the vas deferens remained unaltered. *Omr. omnia præter unguent.* Towards the latter end of September he was made an out-patient. The enlargement and induration of the epididymis and vas deferens were much diminished, but not quite gone.

CASE 5. *Inflammation of the Epididymis and Body of the Testis from blow—Hydrocele.*

John Cobbett, æt. 36, a gardener, admitted October 6, 1830, under Mr. Babington. Oval enlargement, rather broader below than above, half as large again as a full sized egg, in left side of scrotum—tumour transparent. At inferior and external part a hard lump, about the size of a large nut, apparently seated in the globus minor of the epididymis. Testis little enlarged and not at all hard. Pain, of sharp shooting character, in pubes and right loin. No urethral discharge—health good.

Eleven days ago he bruised the testis on the bough of a tree. He suffered pain at the time — in two days afterwards the testis swelled and grew very painful—and the pain continued to augment, as he pursued his usual avocations.

He was purged, leeches were applied over the testicle, a stimulating lotion was employed, and the mouth was rendered slightly sore by the blue pill. On the 20th the epididymis was diminished in size, but there was still some fluid in the tunica vaginalis.

We might adduce many cases of the same description, but we presume that the foregoing will be thought sufficient. It is evident that in acute inflammation of the testis from irritation in the bladder or urethra, the vas deferens and epididymis are, as might à priori be expected, more particularly the parts affected. After the enlargement and induration of the body of the testis have subsided, thickening and consolidation of the vas deferens and epididymis remain, requiring time for their removal. This removal is expedited by support of the part, stimulating lotions on the subsidence of the more acute symptoms, and the subsequent application of the mercurial ointment with camphor, followed by stimulating plasters. Sometimes this chronic induration of the parts in question requires the exhibition of mercury internally, and this is particularly the case when there has been in the first instance, and still remains some fluid effusion into the vaginal tunic. Perhaps, when the testicle has been exposed to mechanical injury, its body suffers more than in hernia humoralis from the causes already mentioned. Yet in several such cases we have seen the vas deferens and epididymis severely affected at first, and remain indurated long afterwards, the body of the testis having apparently recovered itself.

We now proceed to the detail of some cases of a complexion somewhat different from the preceding, to cases of what Sir Astley Cooper has termed simple 'chronic inflammation of the testis;' — and Mr. Brodie, the 'yellow tubercular disease.' It is a subject of great importance. Many a testicle was formerly removed, from the nature of the affection not being well understood, but the diffusion of information throughout the profession is rendering such an unnecessary operation more and more uncommon. For the appearances observed in the dissection of testes thus diseased, we refer to Sir Astley Cooper's work, or the review of it in this journal, and to the papers of Mr. Brodie, in the Medical and Physical Journal.

CASE 6. *Chronic Inflammation apparently from Rheumatism—Mercury—great benefit.*

George Glasspool, æt. 32, a farmer from Berkshire, admitted May 26, 1830, under Mr. Keate.

Left testis enlarged to about the size of an ordinary kidney potatoe—tolerably oval in shape and regular on the surface — hard — not tender on pressure. Cord felt behind, but no distinction of testis and epididymis, at least no separate enlargement of latter, nor induration of the cord. Skin of scrotum rather reddish anteriorly, and adherent to the testis for a space as large as a half-crown piece. On using any exertion, feels a lancinating pain extending from the testis to the 'left kidney,' and accompanied with a sensation of dragging in the loins. No affection of the urine or urinary passages. Health pretty good —face rather sallow.

About twelve months ago, having suffered from what he calls rheumatism in his left upper extremity for three months previously, he found the testicle begin to swell, when the rheumatism gradually disappeared. In two or three days after the commencement of the swelling the lancinating pain came on. Under the application of goulard water and one leech, the swelling, &c. disappeared, and he remained well till the cold weather of last Winter, when the disease returned and has since continued with little let or hindrance; the skin of the scrotum has been adherent for three weeks or a month; has taken no mercury. Has had clap or sore, it is not clear which, three times, the last five years ago, and took calomel on each occasion, but without his mouth becoming affected. At the time when the present complaint commenced he had a large carbuncle on his leg. His mother's father had a large swelling of the testicle, which lasted for many years, until his death, but gave no particular inconvenience. His own brother has a swelling of the testis of six months' standing, and carbuncles.

Leeches were applied two or three times, with purgation, and saline medicines, in order to get rid of the pain about the testis, and some little fever under which the patient laboured. On the 1st June he was ordered *pil. hyd.* gr. v. *nocte maneque.* On the 14th, the mouth being very sore, the pills were omitted, and *ung. hyd. fort.* applied to the testicle. The testis was now much diminished in size and softer, the scrotum not near so adherent, no pain in the loins, cauda epididymis felt distinct and rather harder than the rest. On the 21st, he was compelled by family business to leave the hospital. The enlargement of the testis had still farther subsided, but was not quite dispersed.

CASE 7. *Enlargement of both Testes —Dropsy—Death.*

Thomas Jones, æt. 35, admitted Oct. 27th, 1830, under Mr. Hawkins.

Right testis about three times its natural size, ovoid, narrowest above, smooth on the surface, without distinction of testis and epididymis; to the feel generally hard, except anteriorly and inferiorly, where there is an imperfect sensation communicated of some fluid being situated in the tunica vaginalis; tender on pressure, moderately heavy; vas deferens slightly thickened. Left testis about twice its natural size, ovoid, not so regular on its surface, without distinction of testis and epididymis. Occupying its middle, a hard knotted lump, tender to the touch. Superiorly and inferiorly, a pretty distinct sensation communicated of fluid in the tunica vaginalis. Vas deferens not enlarged. No pain in testes—pain of dull aching kind about right inguinal ring, extending to hips and loins, especially right—pain in left leg. Out of health—aspect sallow—pulse frequent, irritable—tongue foul—bowels habitually costive—belly rather tympanitic—pain on pressure in the hepatic region—emaciation.

Eight or nine years ago had a sore on penis, for which he took mercury, and no secondary symptoms ensued. Has had one or two claps. In India he had symptoms of hepatic character, for which his mouth was made very sore. About ten or twelve months ago he returned to this country, and was for some time in better health, but soon dyspeptic symptoms re-appeared. Three or four months ago he found the right testis enlarged; the left testis swelled afterwards, and soon the pain in the loins succeeded. Has drunk freely of spirits.

He was put upon sarsaparilla and blue pill, the right testis began to diminish in size, but on the 14th November ascites had commenced. It rapidly increased, the legs became anasarcous, and on the 23d December the patient died rather suddenly.

On dissection the liver was found much tuberculated and hardened, with a considerable quantity of fluid in the belly. The disease in the testes was shewn to be chronic inflammation with the yellow tubercular formation. We did not see the examination of the body, but the preparation is in the museum at the hospital.

CASE 8. *Chronic Enlargement of Left Testis—Effusion into the Tunica Vaginalis—Mercury employed.*

Millington Neighbour, æt. 28, a servant, admitted Sept. 15th, 1830, under the care of Mr. Keate.

Enlargement of left testis larger than a Windsor pear, pyriform with the broadest end below—heavy—not painful on pressure, nor when quiet—inconvenient from its weight in walking, when the unpleasant sensation extends to the left loin. Tumour solid behind and below, particularly on the side next the other testis: in front and above more elastic, soft, imperfectly transparent. Scrotum unadherent and tunica vaginalis apparently not thickened. Cord felt rather enlarged, entering the tumour behind and below.

Has had gonorrhœa twice, the first time seven years ago, when the left testis swelled but subsided. Present affection of left testis began two months ago, and for some weeks before that he had had no discharge whatever from the urethra. Swelling commenced from below and gradually mounted upwards. He applied to a surgeon, who made his mouth sore by a *few* pills, without benefit. He went to another surgeon, who gave him a truss, which increased the disease. A country surgeon then said it was hydrocele, tapped it, and let out half a pint of clear fluid, some enlargement of the testis still remaining. In two or three days the tumour regained its former size, and is not increasing.

18th. Pil. hyd. gr. v. bis die.

Oct. 1st. Mouth slightly sore. To-day the hydrocele was punctured with a trocar, and 12 or 14 ounces of very yellow but clear fluid discharged; it coagulated completely by heat and by nitric acid. The testis was felt irregular and enlarged.

*Ung. hyd. c. Camph. nocte maneque, scroto infricand.*

By the 9th the swelling had formed again, but was not quite so large; the testis was felt enlarged, hard, very painful; the epididymis was chiefly affected. In about a week after this he left the hospital at his own desire, in order to return to his situation. The testis was in the same state as at the last report.

CASE 9. *Chronic Inflammation and Abscess in Testis—Mercury—Ulceration of the Scrotum.**

Henry Bishop, ætatis 26, admitted Feb. 17th, 1830, under the care of Mr. Hawkins.

Swelling of the right testicle of considerable size—on its outer side a separate tumefaction feeling softer than the remainder. Pulse very quick—countenance sallow.

Swelling commenced three months ago, and was immediately accompanied by pain and tenderness. It began in the epididymis and thence extended to the body of the testis, which increased in size gradually till three weeks ago, when it became very painful, swelled rapidly, and became soft in one part. At this time he had pain in the loins, white sediment in the urine, and, on examining the urethra, much pain near the membranous part. Four or five days ago the lump on the outside appeared, and has quickly increased. He has been an out-patient; at first he had leeches, &c. then calomel, under which the testis diminished, though the epididymis remained hard and swollen. He took sarsaparilla with the calomel, but it disagreed.

The softer part was punctured, and three ounces of thick pus discharged; the testis was then felt hard and much swollen. He was ordered a dose of calomel and Dover's powder with a purge, and the irritation subsided, whilst the surface of the abscess and the scrotum began to granulate.

23d. *Pil. hyd.* gr. v. *nocte maneque. Dec. sars. c. Oj. ext. sars.* ʒi. *quotid.*

On the 26th there was more pain, which was relieved by matter issuing from another opening into the testis, which was enlarged by a bistoury. On the 4th March the mouth was sore, the testis smaller. On the 13th he was ordered a pint of sarsaparilla, and on the 27th, the mercurial ointment with camphor. On the 3d April, the mouth was more sore; ointment omitted; pill only every night. On the 9th the scrotum had begun to ulcerate, with constitutional disturbance. The sarsa and mercury were discontinued, and he had castor oil, with solution of chloride of soda to the wound. On the 11th the ulceration was spreading.

*H. salin.c. Liq. op. sed.* ♏. x. 4*tis. hor. Haust. opiat. horâ somni.*

* We did not see this patient during his stay in the hospital; the case is taken from Mr. Hawkins' ward-book.

On the 13th, the ulceration was not so rapid in its progress, nor was the surface sloughy, but the testis was extensively exposed, and the edges of the skin were everted; pulse frequent and small. On the 17th the surface of the testis was more healthy; skin undermined and dark coloured; pulse very small and frequent.

*Mist. camph.* ℥iss. *Ammon. carb.* gr. vj. *Tr. op.* ♏. xx. 6*tis hor.* *Solut. acid. nit.* (ʒj. *ad* ℈j.) *ulceri.*

On the 20th, the unguent. opii was applied; on the 27th the edges of the skin were more healthy, and a chloride of soda lotion was used, whilst the patient was put on nitric acid internally. This produced hæmaturia and some hæmorrhage from the bowels, and was omitted on the 6th May. The surface of the ulcer was now granulating kindly, but two sinuses ran into the testis, one to a considerable depth. He was ordered camphor and opium, and in a day or two conium. On the 18th the testis was much more healthy, the sinuses filling up; but the pulse was still frequent. He was made an out-patient.

CASE 10. *Chronic Inflammation of Testis—Abscess—Mercury—Sloughing of Scrotum—Large Mass of Tuberculous Matter removed.*

Isaac Marsh, æt. 32, a labourer from Guilford, admitted May 5th, 1830, under the care of Mr. Brodie.

Solid tumour of right testis, about the size of a Windsor pear, but not quite so long—largest above—irregular in shape, but not very rough on the surface—generally hard and unyielding. At the upper and anterior part a circular slight prominence, more elastic and yielding than the rest—head of epididymis felt distinct—at the upper and external part two or three small circular fistulous openings, through which a thin scanty discharge dribbles out. Tumour generally painful on firm pressure—no pain elsewhere—cord sound—no affection of urinary passages. Health good —appearance rather scrofulous.

Nine months ago he struck the testicle on horse-back, after which it swelled a little and was painful. Six months ago it suddenly became, without obvious cause, more swollen and painful, and has continued to grow worse till within the last month. The abscess at the upper part formed at Christmas, broke, and discharged about a drachm of pus. He had gonorrhœa seven years ago, but was quite cured without injections. Has continued at his work till lately; has been leeched, used lotions, and taken pills for a month; they did not affect his mouth.

*Tinct. Iodinæ*, ♏. x. *t. d.* *Lot. Ammon. Mur. testi.*

21st. *Dec. Sars. c.* ℈j.—*Ext. ejusd.*, ʒij. *Liq. hyd. oxymur.*, ℥ss. *M. quotid.* *Ung. hyd. fort.*, ℥iss. *Ung. pot. hydriod.* ℥ss. *M. testi applic.*

JUNE 25th. The ointment making the scrotum very sore, has been twice changed for the ung. zinci, and resumed as soon as the soreness went off. His mouth is now and has for some time been affected by the oxymuriate, but not severely. Testis more tender, and rather larger—cord a little enlarged—one small inguinal gland enlarged. The fistulous openings discharge more and thicker pus, and the probe passes for an inch or so under the skin of the scrotum. The sinuses were laid open, and granulations were exposed, springing apparently from the outside of the tunica vaginalis; no sinus could be found passing into the interior of the testis.

*Omr. dec. sars.*, *&c.*

*Calom.*, gr. ij. *Opii.*, gr. j., *t. d.*

JULY 8th. Ptyalism—wound of scrotum sloughing—general pyrexia.

*Omr. calomel*, *&c.* *Mist. camph. c. T. opii.*, ♏. x., *t. d.* *Ext. col. c.* gr. v., *o. n.*

16th. Sloughing stopped — nearly whole of right side of scrotum in a state of superficial ulceration — some feverishness and dejection of spirits. *Cataplasma conii.*

On the 23d he was ordered calumba and acids. On the 30th a large mass of tuberculous matter was removed by enlarging one of the ulcerated openings with a bistoury. After this the wound improved, but on the 3d Sept. a probe could be passed deeply down under the integuments of the scrotum, and the sinus was laid freely open. On the 6th he was ordered *pulv. sarsæ*, ℥i, 4*ter die*. On the 30th, the indurated lump had diminished, the sinuses were filling up, the granulations looked healthy, and the health was good.

Oct. 22d. For the last fortnight the testis has remained in a stationary state. To-day Mr. Brodie dissected away the remains of the organ, which was granulating; the bleeding was stopped by blue lint. After this the wound healed perfectly, and the patient was discharged cured in a few weeks.

Case 11. *Chronic Inflammation of both Testes—Sinuses leading into their interior—Mercury.*

John Kinderson, æt. 23, a coachman, admitted July 7, 1830, under the care of Mr. Hawkins.

Enlargement of both testicles. Right about the size of a lemon, but longer —broadest above — with a transverse hardness in its centre, giving it something of an hour-glass form — fluid in the tunica vaginalis above and below. Towards the septum scroti posteriorly and inferiorly the testis feels indurated and knotty. At the inferior part of the scrotum some induration of the superficial parts, which are matted to the lower extremity of the testis; in this indurated part a small circular opening, from which thick yellow matter in small quantity may be pressed; it appears to issue from the testis. Cord sound.

Left testis not quite so large — hard —pretty regular on the surface—painful when handled; the enlargement seems to implicate testis and epididymis, which can be separately felt. At the bottom of the scrotum, much induration of the integuments, which are adherent to the testis, and present a circular fistulous opening, three lines, or more, in diameter, leading into the centre of the gland, and giving issue to a thick yellow pus, Cord sound.

Little pain in either testis, excepting on pressure or exercise — no pain in loins. Health pretty good — appearance strumous—some coppery coloured stains, like those of rupia, on the lower extremities.

Two years and a half ago had gonorrhœa, of which he was cured in three weeks. The eruption (confined to the nates and thighs) appeared first nine months ago, and lasted for a month. Right testis was first affected, and two years ago Mr. Brodie evacuated from the tunica about half a tumbler full of clear fluid. In about three months the testicle began to swell again, and for the last ten months it has been in its present state. Has never had any pain in it. The abscess at the inferior part has existed for three weeks or a month.

Left testis has only been affected for three weeks, the tumefaction first appearing "underneath," where, for the last three years, there was always a sort of a pea. The abscess at the bottom broke about ten days ago. Has not had much pain in the testicle—nothing but poultices have been applied. The disease has not at all affected his sexual powers.

*Pil. hyd.* gr. v. *nocte maneque. Dec. sarsæ.*, ℈ss. *Ext. ejusd.* ℥i. *quotid.*

On the 17th he was ordered two pills at night, and one in the morning, and on the 20th the gums were slightly affected. Both testes were smaller and less painful. On the 21st, only one pill prescribed at night. On the 25th there was pyrexia, with pain in the left testis. The pill and the sarsa were discontinued, and he was ordered infusion of roses and salts every four hours. Leeches were applied three times with much relief, and on the 31st he was enabled to resume the pill and the sarsaparilla. On the 10th there was less pain in both testes; the ulcer over the right was apparently healing—the abscess in the left was enlarged, and discharged healthy pus in greater quantity. He was at this time dismissed on account of irregularity of conduct.

Case 12. *Chronic Inflammation of Testis—Fungus protruding through the Scrotum—Erysipelas—cure.*

Thomas Jones, æt. 20, a carpenter, admitted Sept. 15, 1830, under the care of Mr. Keate.

On right side of scrotum a fungus, larger than two walnuts placed side by side, and of much the same form, projecting an inch or an inch and a half through the skin—surface of fungus displaying a greenish sloughy aspect in some places, large and feeble but otherwise healthy looking granulations in others—it secretes much healthy pus, but no distinct semen—is perfectly free from pain on being handled roughly—is constricted at the part where it passes through the ulcerated scrotal aperture. There does not appear to be much testis in the scrotum, in which the epididymis can be distinguished—cord healthy. Epididymis of other testis rather indurated, enlarged, and painful on pressure. Cord of that testis healthy.

Some pain in right loin; is troubled with nocturnal emissions. Has periosteal nodes on left tibia, and pains in various bones, not aggravated at night. Has on breast and back a cutaneous eruption, consisting of slight crusts, surrounded with a little zone of papulæ; beneath the crusts is a slight depression—no sore throat. Looks rather out of health.

Eighteen months ago had a sore on the penis, for which he took mercury for a month to salivation. A bubo appeared—the sore healed—the bubo was dispersed—and the throat became sore. He took sarsaparilla and thought himself cured. Twelve months ago the testis swelled suddenly and painfully, and *at the same time* the nodes appeared. Had many leeches, and the swelling of the testis diminished, that and the node alternating in pain. Eight months ago the cutaneous eruption appeared. The swelling of the testis increased, and three months ago it broke through the scrotum, when the previous pain was relieved. Has had stricture for four or five years.

17th. *Pil. hyd.* gr. v. *o. n. in* 3 *vices. Cat. lini. Inf. ros. c. Mag. sulph., o. mane.*

20th. *Inf. ros. c. Mag. sulph.* ℥ss. *Tinct. gent. c.* ʒj. *t. d. Omr. alia.*

He was now attacked with erysipelas of the scrotum requiring incisions, ammonia in the first instance, and more powerful support afterwards. In consequence of the erysipelas, two or three abscesses requiring punctures formed in the scrotum, and it was the latter end of October, before the swelling of the scrotum and penis had disappeared. The fungus now looked clean and healthy, and was much diminished in size; it had been for some time dressed with the powdered red precipitate. On the 1st December he was made an out patient. The fungus had entirely disappeared or retired within the scrotum, leaving an ulcer of very minute size, which, indeed, was all but healed. The testis retained to a remarkable degree its natural shape and size, and was not much indurated. He had become very fat, and lost his other ailments. He was directed to take sarsaparilla as an out patient.

CASE 13. *Chronic Fungus of one Testis—Wasting of the other—mercury—Erysipelas—cure.*

Thomas Napp, æt. 29, admitted Jan. 5, 1831, under Mr. Hawkins.

On *left* side of scrotum a chronic fungus of testis, protruding through an ulcerated opening in the integuments with thin inverted edges. Fungus of greyish sloughy appearance on its surface, not at all painful when handled, about the size of a walnut. No inflammation of scrotum around it. Testis felt in the scrotum connected with the fungus, and apparently rather enlarged. Vas deferens a little thickened. No pain in the testis—occasionally a little in the loins. *Right* testis wasted and of very small size. Cicatrix of former ulceration in right groin. Health pretty good—looks rather weakly. Says that he feels the usual sexual desires.

Not quite two years ago had a sore on the penis, and bubo, which suppurated, in the right groin. He took mercury, and in four or five months got rid of his disease, nor has he had secondary symptoms. About five months ago he injured the *left* testis by a blow on a saddle. It inflamed, leeches were applied several times, the acute inflammation subsided, but swelling, pain, and induration remained behind. About two months ago the left side of the scrotum became red and painful. It was poulticed for a week, broke, and gave issue to some cheesy matter first, and then to the present fungus. Of late this has been subsiding rather than increasing; it has been treated by poultices. He has taken no mercury. He knows nothing of the wasting of the right testis.

6th. *Pil. hyd.* gr. v. *nocte maneque. Dec. sars.* ℈ss. *Ext. ejusdem* ʒij. *quotid.*

On the 7th powdered alum was directed to be applied daily to the fungus, and pressure to be made by sticking plaster. On the 11th the mouth was sore; the pill to be repeated only every night. On the 16th, the ptyalism was subsiding; pill night and morning. On the 25th the red precipitate was substituted for the alum, little good having hitherto been affected. On the 28th the mouth was very sore, the fungus diminishing in size, its surface being dry and forming a slough.

*Interm. pilulæ.*

On the 1st Feb. the alum was again resumed, as the fungus was not on the decrease, and this was again changed, on the 5th, for a lotion, consisting of five grains of the white oxyd of arsenic in an ounce of lime-water, with which the surface of the fungus was to be daily washed. After two applications of this lotion, the surface of the fungus became covered with a slough, and erysipelas of the scrotum was established. The erysipelas extended to the nates and thighs, and required ammonia and support. On the 11th day the erysipelas had disappeared. An incision had been made in the scrotum during its continuance, and two or three abscesses, requiring punctures, supervened. After the subsidence of the erysipelas, the fungus speedily diminished in size, being lightly touched every day with the nitrate of silver. At present, March 14, it has completely retired within the scrotum, the ulcerated opening of which has closed and healed. The patient's health, however, is not yet re-established. He is, and has for some time been, taking bark and acid.

Here we must bring this report to a close. We conceive that the cases detailed will give a fair sample of the various stages and aspects of chronic inflammation of the testis. Beginning with what may be regarded as the most simple affection, thickening of the epididymis and vas deferens, left after an attack of acute inflammation, we pass by successive stages to the fungus protruding through the scrotum, or to the conversion of the gland into an inorganized yellow tuberculous mass, incapable of performing any functions, exciting inflammation and suppuration in the scrotum, and requiring, as in case 10, to be removed altogether. We see the value of mercury in this disease, and we also see that here, as elsewhere, it requires to be used with circumspection. In cases 9 and 10, it certainly appeared to produce ulceration and sloughing in the scrotum, and it is to be observed that, in both these instances, there was abscess in the scrotum at the time that it was employed. Whether, under these circumstances, the use of mercury is generally or frequently followed by such consequences, we cannot pretend to determine, as the facts recorded are all we possess that bear upon this point. In case 11, it will be observed that there were sinuses leading into the interior of the testis, and that mercury produced no such mischievous effects, although one testis became inflamed under its use, and required three applications of leeches. We think these cases will serve to demonstrate, that the thickening of the vas deferens and epididymis left by acute inflammation, is much more under the control of mercury than the genuine chronic inflammation of the testis; the former appears to consist merely in the effusion of lymph, the latter, in the deposition of a peculiar, inorganized substance. We might have given many cases, even more illustrative than the preceding, of the power of mercury over this complaint, but our object has rather been to shew its different states and stages, than to put together many specimens of the same description.

We might, perhaps, with even the puny experience that we can boast of, have made more observations than we have done. But our object was to write a report, not an essay; and we have seen so frequently how the study of a great number of facts modifies or overthrows the opinions founded on the observation of a few, that we feel extremely diffident in forming conclusions, and still more cautious of giving them expression.

---

